# Miklós Bodanszky Award Lecture: Advances in the selective targeting of protein phosphatase‐1 and phosphatase‐2A with peptides

**DOI:** 10.1002/psc.3033

**Published:** 2017-09-05

**Authors:** Maja Köhn

**Affiliations:** ^1^ Centre for Biological Signalling Studies (BIOSS) University of Freiburg Schänzlestraße 18 79104 Freiburg Germany; ^2^ Faculty of Biology University of Freiburg Schänzlestrasse 1 79104 Freiburg Germany

**Keywords:** protein phosphatases, allosteric modulation, active site inhibition, peptidomimetics, macrocyclic peptides, microcystin, peptide‐based drug design, phosphatase database DEPOD

## Abstract

Protein phosphatase‐1 and phosphatase‐2A are two ubiquitously expressed enzymes known to catalyze the majority of dephosphorylation reactions on serine and threonine inside cells. They play roles in most cellular processes and are tightly regulated by regulatory subunits in holoenzymes. Their misregulation and malfunction contribute to disease development and progression, such as in cancer, diabetes, viral infections, and neurological as well as heart diseases. Therefore, targeting these phosphatases for therapeutic use would be highly desirable; however, their complex regulation and high conservation of the active site have been major hurdles for selectively targeting them in the past. In the last decade, new approaches have been developed to overcome these hurdles and have strongly revived the field. I will focus here on peptide‐based approaches, which contributed to showing that these phosphatases can be targeted selectively and aided in rethinking the design of selective phosphatase modulators. Finally, I will give a perspective on www.depod.org, the human dephosphorylation database, and how it can aid phosphatase modulator design. © 2017 The Authors. Journal of Peptide Science published by European Peptide Society and John Wiley & Sons Ltd.

**Abbreviations**
Adda: – (2*S*,3*S*,8*S*,9*S*)‐3‐amino‐9‐methoxy‐2,6,8‐trimethyl‐10‐phenyldeca‐4,6‐dienoic acid
*Bpa*: – benzoylphenylalanine
DARPP32: – dopamine and cyclic adenosine monophosphate‐regulated phosphoprotein of molecular weight 32 kDa
DEPOD: – dephosphorylation database
DNA: – deoxyribonucleic acid
G_M_: – muscle form of the PP1 regulatory G subunit
Gm: – PP1 targeting inhibitor peptide derived from G_M_
HeLa: – Henrietta Lax
HIV: – human immunodeficiency virus
I‐2: – Inhibitor‐2
MC: – microcystin
NIPP1: – nuclear inhibitor of PP1
PDP: – PP1 disrupting peptide
pH3: – phosphorylated histone 3
pI‐1: – phosphorylated inhibitor‐1
PIP: – PP1 interacting protein
PP: – protein phosphatase
PP(number)c: – catalytic subunit of a PP
PPP: – phosphoprotein phosphatases
Repo‐Man: – Recruits PP1 onto mitotic chromatin at anaphase protein
RVxF: – single‐letter amino acid code, x = any amino acid excluding proline.

## Introduction: Protein Phosphorylation, Protein Phosphatase‐1 (PP1) and Phosphatase‐2A (PP2A)

Protein serine/threonine kinases and phosphatases are important enzymes governing the process of phosphorylation by adding and removing, respectively, a phosphate group on the hydroxyl groups of serine and threonine amino acids in proteins. Phosphorylation is the most common reversible chemical modification in eukaryotes 1, regulating, for example, the interactions of proteins, their physicochemical properties, their activity, and their stability and degradation 2. The balance between phosphorylation and dephosphorylation is fundamental in many aspects of cell physiology, and disruption of this balance contributes to diseases 3, 4.

**Figure 1:**
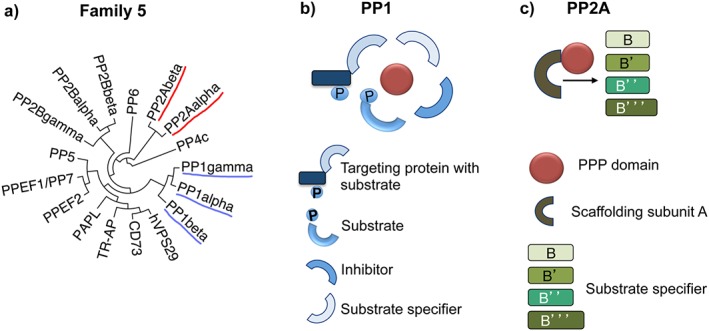
Schematic representation of (a) family 5 phosphatases including the PPPs PP1, PP2A, PP2B, PP4, PP5, PP6, and PP7 (adapted from 5, PP1 and PP2A isoforms underlined), (B) PP1, and (C) PP2A holoenzymes (adapted from 6).

Among the different types of serine/threonine‐specific phosphatases, the phosphoprotein phosphatases (PPPs), which are part of family 5 (Figure 1(a)) 5, are known to dephosphorylate the majority of phospho‐residues on serine and threonine. Their catalytic active sites are highly conserved, which has hampered selective inhibitor design in the past. Within the PPPs, phosphatase‐2A (PP2A) and phosphatase‐1 (PP1) carry out the majority of dephosphorylation reactions, counteracting more than 100 kinases 5. While on first sight it would seem that this fact speaks for PP1 and PP2A being non‐specific, the opposite is actually the case when considering how these enzymes are presented and regulated in cells. They are always bound to regulatory proteins in the form of holoenzymes, altogether accounting for an estimated number of more than 250 PP1 and PP2A holoenzymes 7, 8.

The catalytic subunit of PP1 (PP1c), which exists in the three isoforms PP1α, β, and γ (γ1 and γ2), interacts with more than 200 structurally largely unrelated proteins that determine when and where the phosphatase acts 8. These PP1 interacting proteins (PIPs) function, for example, as substrate‐targeting and/or location‐targeting subunits, substrate‐specifiers, or inhibitors (Figure 1(b)) 8. PP1 holoenzymes function in numerous cellular processes, including cell cycle progression, protein synthesis, muscle contraction, transcription, calcium signaling, and splicing 8, 9, 10. Given their diverse functions, it is not surprising that PP1 holoenzymes also play many roles in a number of diverse pathologies such as heart failure 11, memory loss 12, viral infections 13, type‐2 diabetes 14, and cancer 15.

PP2A holoenzymes are usually trimeric complexes that consist of the catalytic subunit C (PP2Ac), a regulatory B subunit, and the structural subunit A. Regulatory subunit B proteins direct the enzyme to different locations and confer substrate specificity, whereas the subunit A helps to form the holoenzyme complex (Figure 1(c)) 7. These three subunits assemble in a combinatorial fashion to make over 90 PP2A holoenzymes 7. Whereas most of the complexes are trimeric, PP2A can also occur as a dimer with just one A and one C subunit 7. These PP2A holoenzymes regulate, for example, cell proliferation and death, mobility, signaling pathways, mitosis, and DNA damage repair 7, 16. In disease, PP2A holoenzymes play multiple roles in Alzheimer's 17, 18 and cancer 16, 19, 20: PP2A is known to be a tumor suppressor 16. In this regard, it is the A and B subunits that are genetically altered in different types of cancers leading to functional impairment of PP2A, and increased expression of PP2A inhibitory proteins often correlates with cancer aggressiveness and poor prognosis 7. On the other hand, inhibition of PP2A has also been shown to lead to cancer cell death. This is attributed to apoptosis being a consequence of improper cell cycle progression, for which proper PP2A function is crucial. Furthermore, PP2A plays a role in DNA damage repair, and co‐treatment of a PP2A inhibitor with a DNA damaging agent or a DNA repair inhibitor was shown to sensitize cancer cells to chemotherapy‐induced cell death 7.

Considering the diverse roles of PP1 and PP2A in health and disease, it is critical to know the identity and understand the roles of the particular holoenzymes in cellular processes, and to not view them only as PP1 and PP2A catalytic subunits alone.

## Modulation of PP1 or PP2A Activity with Chemical Compounds

Traditionally, inhibiting enzymatic activity has been accomplished by using compounds that target the active site of the enzyme. However, the conserved nature of the active site pocket in PPPs has made it very difficult to achieve selectivity for PP1 or PP2A over the other, and the other PPPs have often been neglected when determining selectivity issues 4. For example, various molecules such as microcystins (MCs), calyculin A, and cantharidin are not specific and are often problematic in their use due to low solubility and cell permeability. Fostriecin is selective toward PP2A over PP1 and PP5, but it is unstable 4. While this continues to be a challenge, new developments now offer the prospect of reaching selective, potent, cell‐active chemical modulators of PP1 and PP2A holoenzyme activity. This has largely been achieved by targeting the holoenzymes instead of the active site of the proteins. Small molecules such as Sephin1, which interferes with PP1 actvity 21, and FTY720193 as well as its analog AAL(S), which were shown to activate PP2A 22, 23, have been groundbreaking for the development of PPP modulators. For a recent overview on these, the reader is referred to reference 4.

In essence, targeting holoenzymes means targeting protein–protein interactions, either on the catalytic subunit or the regulatory protein side, which comes with its own challenges such as the need to disrupt large interaction areas or targeting intrinsically disordered proteins, which are the majority of the interaction partners of PP1 8. Often peptides are the compounds of choice to achieve this, but they also frequently have inherent properties that are not ideal for chemical modulator development, such as low cell permeability, low stability, or low solubility 24, 25. Nevertheless, many examples indicate that these challenges can be overcome to design cell‐active chemical modulators 24, 25.

**Figure 2:**
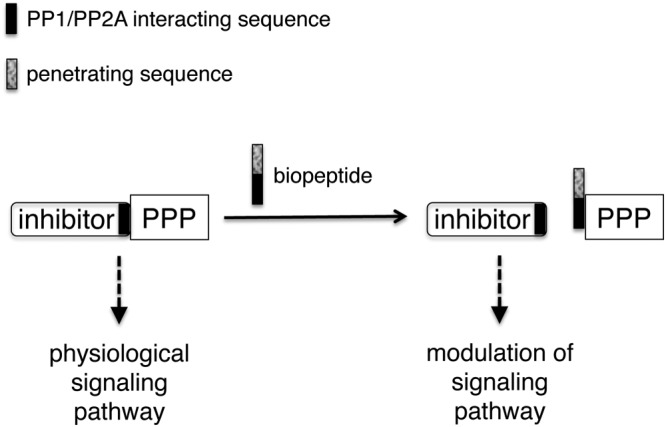
Depiction of the principle of fusing a cell‐penetrating sequence to a PPP‐binding sequence to modulate PPP activity in cells (adapted from 26).

In a proof‐of‐principle study, A. Garcia and his group developed a peptide‐based approach to target PP1 and PP2A with peptides containing a binding‐region and a cell‐penetrating region (Figure 2) 26. To identify the binding sequences that target PP1 and PP2A, they screened cellulose‐bound peptides derived from known interacting proteins. The binding sequences were fused to a cell‐penetrating peptide sequence discovered in the same work. The fusion peptides containing either a binding sequence for PP1 or PP2A were shown to co‐precipitate PP1 and PP2A, respectively, and to induce apoptosis. While the modes of action or the selectivity were not investigated at the time, this study demonstrated that cell‐penetrating peptides targeting these phosphatases at sites other than the catalytic pocket have an effect in cells.

## Targeting the PP1‐Regulatory Protein Interaction with Peptides to Disrupt PP1 Holoenzymes

PP1 contains several bindings sites for PIPs, but the major one that is thought to bind to most PIPs is the so‐called RVxF‐binding site (single‐letter amino acid code, x = any amino acid excluding proline). It is a hydrophobic channel on PP1 to which PIPs containing an RVxF‐type motif bind. This annotation is not strictly defined, as several sequence definitions for this motif have been proposed 27, 28, 29. Both peptides and small molecules have been developed to target the RVxF‐binding site on PP1 8.

The first examples of peptides targeting this site were used to study the binding modes of endogenous PIPs *in vitro*. These were peptides derived from the natural sequences of DARPP32 (dopamine and cyclic adenosine monophosphate‐regulated phosphoprotein of molecular weight 32 kDa) 30 and inhibitor 1 31. A peptide based on spinophilin from residues 438 to 461 disrupted the PP1‐spinophilin holoenzyme in cell lysates at a 100 μM concentration 32. While inhibitor 1 and DARPP32 peptides inhibited PP1 catalytic activity, the spinophilin sequence‐based peptide did not, but it did disrupt holoenzyme formation. This shows that peptides targeting the RVxF‐binding site can be used to modulate PP1 activity in different ways depending on the peptide sequence beside the RVxF‐motif. A first example of applying PP1‐modulating peptides in cells was a peptide that consisted of residues 63 to 75 of the muscle form of the PP1 regulatory G subunit, G_M_ 33, 34. This peptide contained the RVxF‐motif and disrupted PP1 holoenzymes but not its catalytic activity and was termed PP1 targeting inhibitor peptide Gm 34. For cellular uptake, it needed to be dialyzed into cells through a patch pipette 34. Further 4 to 13‐mer peptides based on the sequence of G_M_ were shown to activate PP1 and disrupt the complex between PP1 and DARPP32 using low micromolar concentrations *in vitro*, demonstrating that a tetrapeptide (Ac‐RVSF‐*N*Me) could be sufficiently potent, but neither their activity in cells nor their selectivity was tested 35. Finally, expression of an 82‐mer fragment of the central domain of nuclear inhibitor of PP1 (NIPP1) was reported to disrupt the interaction between HIV (human immunodeficiency virus)‐1 trans‐activator of transcription protein and PP1, inhibiting HIV‐1 replication in cells 13. No other tested PIPs were affected in this context, which could be due to the relatively low affinity of trans‐activator of transcription protein to PP1 that was blocked, whereas endogenous holoenzymes that have a higher affinity were not disrupted 8, 13, 36.

**Figure 3:**
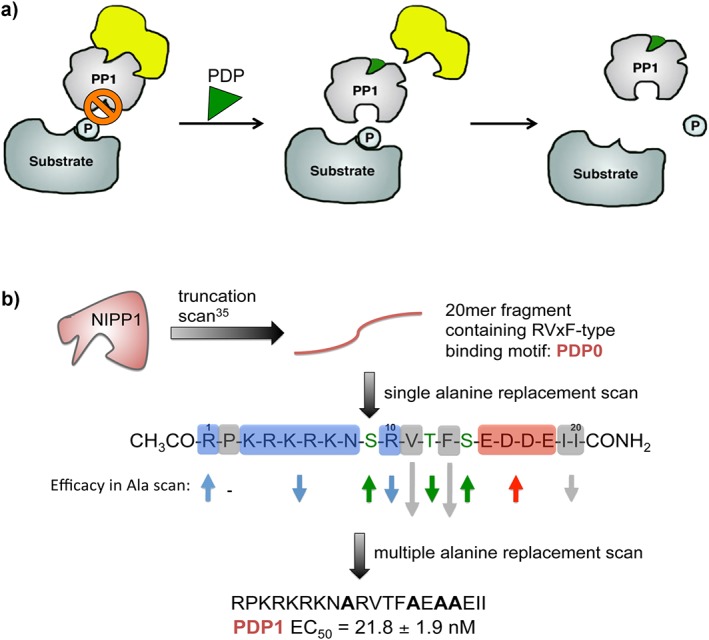
Mode of action of PDPs (a) and their design (b). (a) PDPs disrupt PP1 holoenzymes by binding to the RVxF‐binding site on PP1c and competing with endogenous PIPs. The released catalytic subunit can dephosphorylate nearby substrates. For testing the potency of the peptides, the release of the inhibitory protein I‐2 (yellow) from PP1c (gray) was measured by the resulting increase in PP1c phosphatase activity on the dephosphorylation of [31]P‐labeled phosphorylated glycogen phosphorylase *a* (blue substrate). (B) Based on a 20‐mer fragment of NIPP1 and PDP0, single and multiple alanine replacement scans led to the design of PDP1.

Building on these findings, in collaboration with Mathieu Bollen, KU Leuven, Belgium, we set out to develop peptides that would target the RVxF‐binding site on PP1, modulate PP1 activity selectively in cells, and be cell penetrating as well as proteolytically stable, and we called these PP1‐disrupting peptides (PDPs) 24. The design was based on data from the Bollen lab, which identified the 20‐mer peptide NIPP1 (191–210) as being able to compete for PP1c inhibition with full‐length NIPP1, as well as with the endogenous protein inhibitors phospho‐inhibitor‐1 (pI‐1) and inhibitor‐2 (I‐2) 37. The first peptide following this design was termed PDP0. Alanine scans based on PDP0 were conducted with different peptides, where either single or multiple amino acids were replaced by alanine. These were screened by measuring the decrease of inhibition of PP1c by I‐2 through the resulting increase in PP1c phosphatase activity on the dephosphorylation of ^32^P‐labeled phosphorylated glycogen phosphorylase *a* (starting at 30% and releasing to 100% phosphatase activity) (Figure 3(a)). Truncation of PDP0 led to a reduction in potency, revealing that the *N*‐terminal polybasic sequence and also the *C*‐terminal isoleucines (ΘΘ‐motif 38) were important for binding. This approach led to an optimized sequence, PDP1, which showed nanomolar potency in the assay (Figure 3(b)). Further attempts to enhance the potency and stability by cyclizing the peptides were not successful, which suggests that the reported conformational flexibility of the RVxF‐motif and the surrounding sequence 39 is beneficial for binding.

While cells did not take up PDP1, extending the polybasic sequence by sequential addition of lysine and arginine residues (RRK) to the *N*‐terminus provided the cell penetrating PDP2. Subsequently, we showed that PDPs do not bind to PP2A and that they disrupt PP1 holoenzymes, the extent of which is dependent on the affinity of the PP1–PIP interaction. The crystal structure of the PP1–PDP2 complex was provided as proof of direct interaction *in vitro*. Furthermore, we designed PDP3 based on PDP2, which carried the photo‐crosslinkable amino acid 4‐benzoylphenylalanine (*Bpa*) instead of alanine adjacent to the phenylalanine of the RVxF‐motif. This sequence was crosslinked to PP1c in cell lysates, indicating direct interaction under these conditions. Finally, we showed that PDP3, but not PDP2, induces PP1 activity in cells by studying phosphorylated histone 3 (pH3) dephosphorylation by PP1 in mitosis. PDP2 is less stable in cells than PDP3 and is therefore likely degraded before it can act in the rather long time frame of this process. During mitosis, the PIP Repo‐Man guides PP1 to its substrate pH3. Knockdown of Repo‐Man prevents the guidance, and PP1 is not able to dephosphorylate pH3. PDP3 rescued this phenotype, suggesting that it binds to PP1 and releases the PDP3‐bound PP1 catalytic subunit that can then dephosphorylate nearby substrates 24. The scope of substrates dephosphorylated by this PP1–PDP3 complex is at this point unclear, as PP1 could act promiscuously or specifically depending on the substrates, their (local) availability, and regulation. This is currently a very difficult question to address given that PP1 is assumed to dephosphorylate the majority of proteins on serine and threonine in eukaryotic cells 39, that comparably only few substrates are known, and that it is unclear which substrates are dephosphorylated redundantly by different PPPs 5.

We used the PDPs to study the acute effect of PP1 activity on calcium signaling 40. Treatment of HeLa cells with PDP2 and PDP3 triggered calcium oscillations in a concentration‐dependent manner within seconds, visualized by the ratiometric Fura‐2 dye. Calcium spikes triggered by PDP3 displayed a higher frequency than the ones for PDP2. While washing out of PDP2 abolished the calcium signals, the cells treated with PDP3 followed by washing continued calcium spiking. Both observations are in agreement with the lower stability of PDP2 24, which is still able to trigger calcium signals but to a lower extent and reversibly, revealing the intriguing possibilities of fine‐tuning the cellular response toward peptides by varying their proteolytic stability. We went on to show that the released calcium originated from the endoplasmic reticulum and that the inositol 1,4,5‐trisphosphate receptor was involved in the release 40. Finally, we demonstrated that the first calcium spike was triggered by PP1, revealing that elevated PP1 activity leads to upregulation of calcium signaling and that PDPs did not activate PP2A and PP2B activity, confirming their selectivity and demonstrating the applicability of these chemical tools to address questions concerning PP1 biology 40.

Subsequent to our work, 4 to 11‐mer peptides based on the *N*‐terminal domain of inhibitor 1 that disrupt the interaction between PP1 and pI‐1 as well as PP1 and I‐2 were reported 41. The most active peptides were a nonapeptide (SPRKIQFTV) and a hexapeptide (RKIQFT), containing a variation (RKIQF) of the RVxF‐motif, both with low micromolar potencies *in vitro* in an assay measuring the decrease of inhibition of PP1c by pI‐1 (from 50 to 100% PP1 activity). In order to check the activity of the peptides inside cells, a polyarginine sequence was attached for cellular uptake. Treatment of cardio myocytes with 10 μM of the polyArg‐nonapeptide led to the reduction of isoprenaline‐induced phosphorylation levels of phospholamban on Ser16 but not of cardiac troponin inhibitor on Ser23/24. Selectivity over other phosphatases was not tested 41.

Taken together, when targeting the RVxF‐binding site on PP1 with peptides, different sets of PP1 holoenzymes can be disrupted depending on the potency of the compounds, the affinity of the PP1–PIP interaction, and the cellular context. The sequence adjacent to the RVxF‐motif can be varied to either inhibit PP1, which means blocking the active site in addition to the RVxF‐site, or to release the free catalytic subunit, leading to dephosphorylation of nearby substrates.

## Efficient Synthesis of a Complex Macrocyclic Peptide Renders an Unselective Active Site Inhibitor Selective

**Figure 4:**
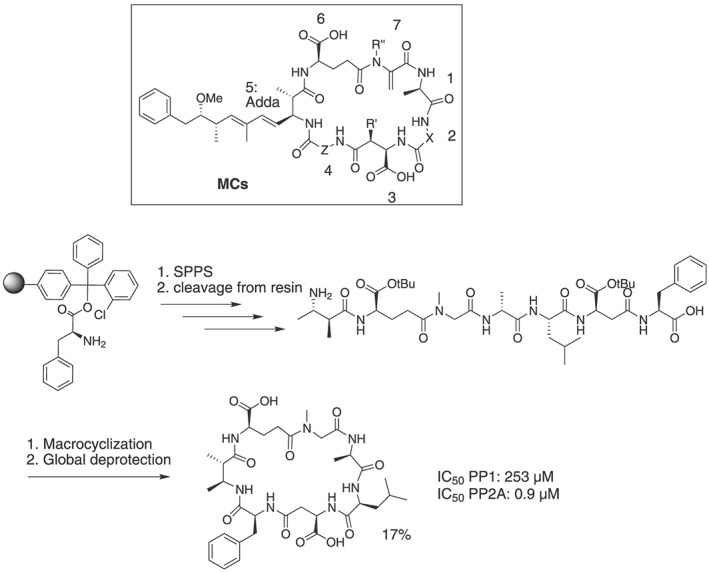
The general structure of MCs and their synthetic procedure, exemplified by the most selective derivative, are shown. The potency toward PP1 and PP2A of this derivative is included. Adda = (2*S*,3*S*,8*S*,9*S*)‐3‐amino‐9‐methoxy‐2,6,8‐trimethyl‐10‐phenyldeca‐4,6‐dienoic acid 43.

Many PPP inhibitors are natural toxins, and they are usually not selective toward a certain PPP 4. With the exception of norcantharidin and similar compounds, these natural toxins have complex chemical structures and are challenging to synthesize, which has hampered their structure–activity relationship studies in the past 42. Peptide natural toxins that inhibit PPPs include the MCs, which are cyclic heptapeptides with the typical structure depicted in Figure 4 43. Around 100 different MCs, so‐called congeners, are known, and they mostly differ in the amino acid composition in positions 2 and 4 44. MCs indiscriminately target PPP active sites, which are highly conserved, and are highly potent inhibitors with potencies in the picomolar to nanomolar range. The so‐called ‘Adda’ side chain at position 5 was reported to be required for binding (Figure 4) 42. We reasoned that given that the general structure of the MCs covers a larger area in and around the PPP active sites, small changes in the molecules might give insights into determinants for selectivity between PP1 and PP2A 43. We therefore set out to synthesize several MC derivatives and to study their structure–activity relationship toward PP1 and PP2A. However, since MCs require multi‐step syntheses that result in low yields, we first designed an efficient solid/solution‐phase synthetic approach and simplified the molecules. Besides other minor changes, we replaced the long lipophilic tail in the beta position of the Adda amino acid at position 5 with shorter tails and synthesized derivatives with or without the α‐methyl group, as this residue had not been investigated for its role in binding before. Our synthetic approach included the assembly of the linear peptide on resin, cleavage, and then macrocyclization in solution followed by global deprotection (Figure 4), leading to good overall yields of the final compounds and enabling the synthesis of 11 MC analogs. We discovered that the α‐methyl group on the backbone of the amino acid in position 5 is highly beneficial for binding, likely replacing a water molecule in a small hydrophobic pocket. Most surprisingly and against previous observations stating that the lipophilic side chain in Adda is required for binding, we obtained sub‐micromolar inhibitors for PP2A that included the α‐methyl group but not the lipophilic tail in position 5. Finally, we discovered inhibitors that were more than 250‐fold selective toward PP2A over PP1 (Figure 4), which is an unprecedented value, obtained through chemical modification of a natural toxin. We showed that this selectivity is largely based on a span of residues in PP1 and PP2A that is not completely conserved: Y(265)CYRC(269) in PP2A aligns with Y(272)CGEF(276) in PP1. While R268 in PP2A can form a hydrogen bond with a carbonyl group on the MC analog, E275 in PP1 cannot. In addition, C269 in PP2A is less bulky than F276 in PP1, helping to accommodate the sterics of MC in the binding pocket 43. Interestingly, this stretch is conserved between PP2A, and PP4 and PP6, which are the closest homologs to PP2A; thus, the MC analogs could also favor these PPPs. However, these residues are not conserved in PP5 (YCDQM), adding another opportunity to achieve selectivity for this phosphatase. PP3 and PP7 are not strongly inhibited by MCs due to unique regions in their catalytic domains 4. Together with studies on non‐peptidic toxins and small molecules 4, 42, 45, these results demonstrate that selective inhibitors targeting the active site of PPPs can be achieved.

## depod.org: A Database Aiding Phosphatase Modulator Design

**Figure 5:**
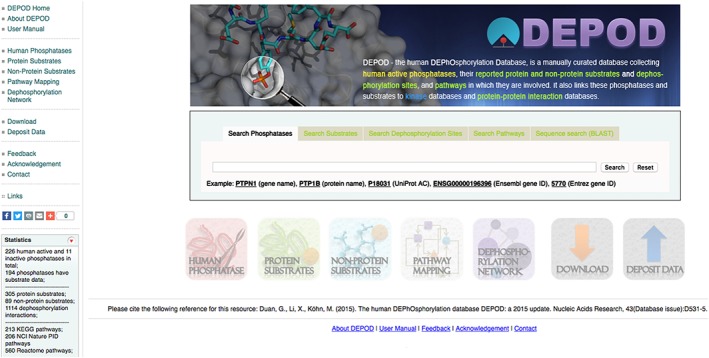
Web interface of www.depod.org.

When we began our studies, data on phosphatases and their substrates and interacting proteins used to be scattered around in the literature. There was no comprehensive database to aid phosphatase studies, so we began to develop the human DEPhOsphorylation Database (DEPOD; www.depod.org). In collaboration with Janet Thornton, European Bioinformatics Institute, UK, we built the database to include all human phosphatases, their substrates (protein and non‐protein), links to the corresponding kinases in other databases, links to small molecule inhibitors (ChEMBL database), and information on the involvement of phosphatases and substrates in cellular pathways 5. We furthermore added interacting proteins (other than substrates) to the database 46. It is searchable for all internal data (phosphatases, substrates, pathways, dephosphorylation sites, and sequences) (Figure 5) and offers an interactive network to visualize phosphatase–substrate interactions. We analyzed the data in several ways; for example, we did a structural evolutionary analysis that led to the re‐classification of phosphatases showing previously unrecognized relationships, and we looked for substrate specificity on the protein domain and sequence levels 5.

DEPOD offers valuable information for the design of chemical modulators of phosphatases. It gives information on substrates and pathways that would be affected upon chemical modulation of a certain phosphatase, and it provides information on direct interactors that could be used to design peptidic modulators of phosphatases, as outlined above. In the case of substrate‐based inhibitors, one can directly analyze which other phosphatases might be affected by the inhibitor in case they recognize the same substrate that was the basis for the compound. Together with other databases (for an overview see reference 4), DEPOD is a valuable resource for phosphatase research and modulator design.

## Perspectives

The complex regulation of PP1 and PP2A is not only a challenge but also offers the possibility to take advantage of the several ways that PP1 and PP2A interact with their regulatory proteins and substrates in order to interfere with their roles in certain cellular pathways and, by extension, various diseases. This disruption can be achieved by targeting either the catalytic subunits or the holoenzymes. Exciting new structural insights into the interactions of different PPPs with their regulatory proteins will strongly support modulator design 38. Peptides play a special role in these strategies: they allow the targeting of larger surfaces and were shown to impart selectivity to modulators targeting these phosphatases. Addressing specific holoenzymes or their cellular localization and distinguishing between the different PPPs still remain major challenges. Given the recent advances in utilizing peptides as the basis for modulator design and the insights obtained so far from employing these strategies, peptides will undoubtedly aid in overcoming these challenges and continue to play an important role in the progression of our understanding of phosphatase biology.

## References

[1] Khoury GA , Baliban RC , Floudas CA . Proteome‐wide post‐translational modification statistics: frequency analysis and curation of the swiss‐prot database. Sci. Rep. 2011; 1: srep00090. 10.1038/srep00090 PMC3201773 22034591

[2] Nishi H , Hashimoto K , Panchenko A . Phosphorylation in Protein‐Protein Binding: Effect on Stability and Function. Structure 2011; 19: 1807–1815. 22153503 10.1016/j.str.2011.09.021 PMC3240861

[3] Fontanillo M , Köhn M . Phosphatases: Their roles in cancer and their chemical modulators. Adv. Exp. Med. Biol. 2016; 917: 209–240. 27236558 10.1007/978-3-319-32805-8_10

[4] Fahs S , Lujan P , Köhn M . Approaches to study phosphatases. ACS Chem. Biol. 2016; 11: 2944–2961. 27700050 10.1021/acschembio.6b00570

[5] Li X , Wilmanns M , Thornton J , Köhn M . Elucidating human phosphatase‐substrate networks. Sci. Signal. 2013; 6: rs10. 10.1126/scisignal.2003203 23674824

[6] DeMunter S , Köhn M , Bollen M . Challenges and opportunities in the development of protein phosphatase‐directed therapeutics. ACS Chem. Biol. 2013; 8: 36–45. 23214403 10.1021/cb300597g

[7] Lambrecht C , Haesen D , Sents W , Ivanova E , Janssens V . Structure, regulation and pharmacological modulation of PP2A phosphatases. Methods Mol. Biol. 2013; 1053: 283–305. 23860660 10.1007/978-1-62703-562-0_17

[8] Chatterjee J , Köhn M . Targeting the untargetable: recent advances in the selective chemical modulation of protein phosphatase‐1 activity. Curr. Opin. Chem. Biol. 2013; 17: 361–368. 23647984 10.1016/j.cbpa.2013.04.008

[9] Cohen PT . Protein phosphatase 1‐targeted in many directions. J. Cell Sci. 2002; 115: 241–256. 11839776 10.1242/jcs.115.2.241

[10] Ceulemans H , Bollen M . Functional diversity of protein phosphatase‐1, a cellular economizer and reset button. Physiol. Rev. 2004; 84: 1–39. 14715909 10.1152/physrev.00013.2003

[11] Nicolaou P , Hajjar RJ , Kranias EG . Role of protein phosphatase‐1 inhibitor‐1 in cardiac physiology and pathophysiology. J. Mol. Cell Cardiol. 2009; 47: 365–371. 19481088 10.1016/j.yjmcc.2009.05.010 PMC2716438

[12] Genoux D , Haditsch U , Knobloch M , Michalon A , Storm D , Mansuy IM . Protein phosphatase 1 is a molecular constraint on learning and memory. Nature 2002; 418: 970–975. 12198546 10.1038/nature00928

[13] Ammosova T , Yedavalli VR , Niu X , Jerebtsova M , Van Eynde A , Beullens M , Bollen M , Jeang KT , Nekhai S . Expression of a protein phosphatase 1 inhibitor, cdNIPP1, increases CDK9 threonine 186 phosphorylation and inhibits HIV‐1 transcription. J. Biol. Chem. 2011; 286: 3798–3804. 21098020 10.1074/jbc.M110.196493 PMC3030381

[14] Xia J , Scherer SW , Cohen PT , Majer M , Xi T , Norman RA , Knowler WC , Bogardus C , Prochazka M . A common variant in PPP1R3 associated with insulin resistance and type 2 diabetes. Diabetes 1998; 47: 1519–1524. 9726244 10.2337/diabetes.47.9.1519

[15] Nuytten M , Beke L , Van Eynde A , Ceulemans H , Beullens M , Van Hummelen P , Fuks F , Bollen M . The transcriptional repressor NIPP1 is an essential player in EZH2‐mediated gene silencing. Oncogene 2008; 27: 1449–1460. 17724462 10.1038/sj.onc.1210774

[16] Westermarck J , Hahn WC . Multiple pathways regulated by the tumor suppressor PP2A in transformation. Trends Mol. Med. 2008; 14: 152–160. 18329957 10.1016/j.molmed.2008.02.001

[17] Sontag E , Luangpirom A , Hladik C , Mudrak I , Ogris E , Speciale S , White CL, 3rd. Altered expression levels of the protein phosphatase 2A ABαC enzyme are associated with Alzheimer disease pathology. J. Neuropathol. Exp. Neurol. 2004; 63: 287–301. 15099019 10.1093/jnen/63.4.287

[18] Vogelsberg‐Ragaglia V , Schuck T , Trojanowski JQ , Lee VM . PP2A mRNA expression is quantitatively decreased in Alzheimer's disease hippocampus. Exp. Neurol. 2001; 168: 402–412. 11259128 10.1006/exnr.2001.7630

[19] Mumby M . PP2A: unveiling a reluctant tumor suppressor. Cell 2007; 130: 21–24. 17632053 10.1016/j.cell.2007.06.034

[20] Sangodkar J , Farrington CC , McClinch K , Galsky MD , Kastrinsky DB , Narla G . All roads lead to PP2A: exploiting the therapeutic potential of this phosphatase. FEBS Journal 2016; 283: 1004–1024. 26507691 10.1111/febs.13573 PMC4803620

[21] Das I , Krzyzosiak A , Schneider K , Wrabetz L , D'Antonio M , Barry N , Sigurdardottir A , Bertolotti A . Preventing proteostasis diseases by selective inhibition of a phosphatase regulatory subunit. Science 2015; 348: 239–242. 25859045 10.1126/science.aaa4484 PMC4490275

[22] Yang Y , Huang Q , Lu Y , Li X , Huang S . Reactivating PP2A by FTY720 as a Novel therapy for AML with CKIT tyrosine kinase domain mutation. J. Cell. Biochem. 2012; 113: 1314–1322. 22109829 10.1002/jcb.24003

[23] Toop H , Dun M , Ross B , Flanagan H , Verrills N , Morris J . Development of novel PP2A activators for use in the treatment of acute myeloid leukaemia. Org. Biomol. Chem. 2016; 14: 4605–4616. 27102578 10.1039/c6ob00556j

[24] Chatterjee J , Beullens M , Sukackaite R , Qian J , Lesage B , Hart DJ , Bollen M , Köhn M . Development of a peptide that selectively activates protein phosphatase‐1 in living cells. Angew. Chem. Int. Ed. 2012; 51: 10054–10059. 10.1002/anie.201204308 PMC3531619 22962028

[25] Fosgerau K , Hoffmann T . Peptide therapeutics: current status and future directions. Drug Discov. Today 2015; 20: 122–128. 25450771 10.1016/j.drudis.2014.10.003

[26] Guergnon J , Dessauge F , Dominguez V , Viallet J , Bonnefoy S , Yuste VJ , Mercereau‐Puijalon O , Cayla X , Rebollo A , Susin SA , Bost PE , Garcia A . Use of penetrating peptides interacting with PP1/PP2A proteins as a general approach for a drug phosphatase technology. Mol. Pharmacol. 2006; 69: 1115–1124. 16387795 10.1124/mol.105.019364

[27] Wakula P , Beullens M , Ceulemans H , Stalmans W , Bollen M . Degeneracy and function of the ubiquitous RVXF motif that mediates binding to protein phosphatase‐1. J. Biol. Chem. 2003; 278: 18817–18823. 12657641 10.1074/jbc.M300175200

[28] Hendrickx A , Beullens M , Ceulemans H , Den Abt T , Van Eynde A , Nicolaescu E , Lesage B , Bollen M . Docking Motif‐Guided Mapping of the Interactome of Protein Phosphatase‐1. Chem. Biol. 2009; 16: 365–371. 19389623 10.1016/j.chembiol.2009.02.012

[29] Meiselbach H , Sticht H , Enz R . Structural analysis of the protein phosphatase 1 docking motif: Molecular description of binding specificities identifies interacting proteins. Chem. Biol. 2006; 13: 49–59. 16426971 10.1016/j.chembiol.2005.10.009

[30] Kwon YG , Huang HB , Desdouits F , Girault JA , Greengard P , Nairn AC . Characterization of the interaction between DARPP‐32 and protein phosphatase 1 (PP‐1): DARPP‐32 peptides antagonize the interaction of PP‐1 with binding proteins. Proc. Natl. Acad. Sci. U. S. A. 1997; 94: 3536–3541. 9108011 10.1073/pnas.94.8.3536 PMC20474

[31] Endo S , Zhou X , Connor J , Wang B , Shenolikar S . Multiple structural elements define the specificity of recombinant human inhibitor‐1 as a protein phosphatase‐1 inhibitor. Biochemistry 1996; 35: 5220–5228. 8611507 10.1021/bi952940f

[32] Yan Z , Hsieh‐Wilson L , Feng J , Tomizawa K , Allen PB , Fienberg AA , Nairn AC , Greengard P . Protein phosphatase 1 modulation of neostriatal AMPA channels: regulation by DARPP‐32 and spinophilin. Nat. Neurosci. 1999; 2: 13–17. 10195174 10.1038/4516

[33] Johnson DF , Moorhead G , Caudwell FB , Cohen P , Chen YH , Chen MX , Cohen PT . Identification of protein‐phosphatase‐1‐binding domains on the glycogen and myofibrillar targetting subunits. Eur. J. Biochem. 1996; 239: 317–325. 8706735 10.1111/j.1432-1033.1996.0317u.x

[34] Westphal RS , Tavalin SJ , Lin JW , Alto NM , Fraser ID , Langeberg LK , Sheng M , Scott JD . Regulation of NMDA receptors by an associated phosphatase‐kinase signaling complex. Science 1999; 285: 93–96. 10390370 10.1126/science.285.5424.93

[35] Tappan E , Chamberlin AR . Activation of protein phosphatase 1 by a small molecule designed to bind to the enzyme's regulatory site. Chem. Biol. 2008; 15: 167–174. 18291321 10.1016/j.chembiol.2008.01.005

[36] Ammosova T , Jerebtsova M , Beullens M , Lesage B , Jackson A , Kashanchi F , Southerland W , Gordeuk VR , Bollen M , Nekhai S . Nuclear targeting of protein phosphatase‐1 by HIV‐1 Tat protein. J. Biol. Chem. 2005; 280: 36364–36371. 16131488 10.1074/jbc.M503673200

[37] Beullens M , Van Eynde A , Vulsteke V , Connor J , Shenolikar S , Stalmans W , Bollen M . Molecular Determinants of Nuclear Protein Phosphatase‐1 Regulation by NIPP‐1. J. Biol. Chem. 1999; 274: 14053–14061. 10318819 10.1074/jbc.274.20.14053

[38] Peti W , Page R . Strategies to make protein serine/threonine (PP1, calcineurin) and tyrosine phosphatases (PTP1B) druggable: achieving specificity by targeting substrate and regulatory protein interaction sites. Bioorg. Med. Chem. 2015; 23: 2781–2785. 25771485 10.1016/j.bmc.2015.02.040 PMC4451382

[39] Bollen M , Peti W , Ragusa MJ , Beullens M . The extended PP1 toolkit: designed to create specificity. Trends Biochem. Sci. 2010; 35: 450–458. 20399103 10.1016/j.tibs.2010.03.002 PMC3131691

[40] Reither G , Chatterjee J , Beullens M , Bollen M , Schultz C , Köhn M . Chemical activators of protein phosphatase‐1 induce calcium release inside intact cells. Chem. Biol. 2013; 20: 1179–1186. 23972940 10.1016/j.chembiol.2013.07.008

[41] Sotoud H , Borgmeyer U , Schulze C , El‐Armouche A , Eschenhagen T . Development of phosphatase inhibitor‐1 peptides acting as indirect activators of phosphatase 1. Naunyn. Schmiedebergs. Arch. Pharmacol. 2015; 388: 283–293. 25416155 10.1007/s00210-014-1065-2

[42] Sakoff JA , McCluskey A . Protein Phosphatase Inhibition: Structure Based Design. Towards New Therapeutic Agents. Curr. Pharm. Des. 2004; 10: 1139–1159. 15078146 10.2174/1381612043452686

[43] Fontanillo M , Zemskov I , Häfner M , Uhrig U , Salvi F , Simon B , Wittmann V , Köhn M . Synthesis of highly selective submicromolar microcystin‐based inhibitors of protein phosphatase (PP)2A over PP1. Angew. Chem. Int. Ed. 2016; 55: 13985–13989. 10.1002/anie.201606449 PMC5113787 27723199

[44] Puddick J , Prinsep MR , Wood SAF , Kaufononga SA , Cary SC , Hamilton DP . High levels of structural diversity observed in microcystins from Microcystis CAWBG11 and characterization of six new microcystin congeners. Mar. Drugs 2014; 12: 5372–5395. 25402827 10.3390/md12115372 PMC4245536

[45] Swingle M , Volmar CH , Saldanha SA , Chase P , Eberhart C , Salter EA , D'Arcy B , Schroeder CE , Golden JE , Wierzbicki A , Hodder P , Honkanen RE . An Ultra‐High‐Throughput Screen for Catalytic Inhibitors of Serine/Threonine Protein Phosphatases Types 1 and 5 (PP1C and PP5C). J. Biomol. Screen. 2017; 22: 21–31. 10.1177/1087057116668852 PMC8041090 27628691

[46] Duan G , Li X , Köhn M . The human DEPhOsphorylation database DEPOD: a 2015 update. Nucleic Acids Res. 2015; 43: Database issue D531–D535. 10.1093/nar/gku1009 PMC4383878 25332398

